# Cost-effectiveness of tirofiban for acute ischemic stroke without large or medium-sized vessel occlusion: A Markov modelling analysis from the Chinese and United States perspectives

**DOI:** 10.1371/journal.pone.0297939

**Published:** 2024-02-16

**Authors:** Li Wang, Yuhong Zeng, Limei Zhou, Ping Xu, Xianbin Guo, Yu Xie, Junxiu Cai, Min Pan, Jie Tang, Qingtao Gong, Rong Su, Yan Liu, Yake Lou

**Affiliations:** 1 Department of Neurology, Zigong Third People’s Hospital, Zigong, China; 2 Department of Epidemiology, College of Preventive Medicine, Army Medical University (Third Military Medical University), Chongqing, People’s Republic of China; 3 Evidence-based Medicine and Clinical Epidemiology Center, Army Medical University (Third Military Medical University), Chongqing, People’s Republic of China; 4 Department of Cardiology, The Second Affiliated Hospital of Chongqing Medical University, Chongqing, China; Bach Mai Hospital, VIET NAM

## Abstract

**Background:**

The RESCUE BT2 trial recently showcased the efficacy of tirofiban in treating acute ischemic stroke (AIS) without large or medium-sized vessel occlusion. To further assess the value of tirofiban from the perspectives of Chinese and US healthcare system, a study was conducted to evaluate its cost-effectiveness.

**Methods:**

A hybrid model, integrating a short-term decision tree with a long-term Markov model, was developed to assess cost-effectiveness between tirofiban and aspirin for stroke patients without large or medium-sized vessel occlusion. Efficacy data for tirofiban was sourced from the RESCUE BT2 trial, while cost information was derived from published papers. Outcomes measured included respective cost, effectiveness, and incremental cost-effectiveness ratio (ICER). We conducted a one-way sensitivity analysis to assess the robustness of the results. Additionally, we performed probabilistic sensitivity analysis (PSA) through 10,000 Monte Carlo simulations to evaluate the uncertainties associated with the results.

**Results:**

The study revealed that tirofiban treatment in AIS patients without large or medium-sized vessel occlusion led to a considerable reduction of 2141 Chinese Yuan (CNY) in total cost, along with a lifetime gain of 0.14 quality-adjusted life years (QALYs). In the US settings, tirofiban also exhibited a lower cost ($197,055 versus $201,984) and higher effectiveness (4.15 QALYs versus 4.06 QALYs) compared to aspirin. One-way sensitivity analysis revealed that post-stroke care costs and stroke utility had the greatest impact on ICER fluctuation in both Chinese and US settings. However, these variations did not exceed the willingness-to-pay threshold. PSA demonstrated tirofiban’s superior acceptability over aspirin in over 95% of potential scenarios.

**Conclusion:**

Tirofiban treatment for AIS without large or medium-sized vessel occlusion appeared dominant compared to aspirin in both China and the US.

## Introduction

Stroke is one of the leading causes of global mortality and a major contributor to serious, long-term disability [1, 2]. The data from Global Burden of Disease (GBD) 2019 showed that there were 101 million prevalent stroke, 6.55 million deaths and 143 million disability-adjusted life years (DALYs) from stroke in 2019 worldwide, with the bulk of the burden in the low- and middle-income counties [3]. In 2019, stroke affected more than 20 million people in China, leading to about 2 million deaths [4]. The medical expenditure was 20.71 billion Chinese Yuan (CNY), with an average annual growth rate of 24.96% [5]. The burden of stroke is also significant in the United States (US), with more than 7 million stroke suffers and an estimated one American experiencing a stroke every 40 seconds, and the direct and indirect costs of stroke in the US were about $45.5 billion in 2014–2015 [6].

Among the incident stroke cases, ischemic strokes accounted for two-thirds, with the remainder composed of intracerebral and subarachnoid hemorrhages [3]. Restoring or improving perfusion in the ischemic area is the core treatment for acute ischemic stroke (AIS), and intravenous thrombolysis (IVT) with recombinant tissue plasminogen activator (rt-PA) and endovascular thrombectomy (EVT) are two main therapies to save ischemic penumbra [7]. However, the narrow time window (usually <4.5 hours or up to 9 hours in selected patients) and the strict indications of IVT limit its widespread application, [8–11] while the EVT is only effective in patients with large vessel occlusion [12]. Tirofiban, a nonpeptide selective glycoprotein IIb/IIIa receptor inhibitor, has been used to treat patients with acute coronary syndrome, [13] and now studies have gradually found that it may also benefit patients with ischemic stroke [14–16]. Recently, a multicenter clinical trial in China, RESCUE BT2 (Tirofiban for Stroke without Large or Medium-Sized Vessel Occlusion), found that tirofiban was associated with a higher likelihood of getting a better modified Rankin scale (mRS) score compared with low-aspirin, [17] which bring new opportunities for AIS patients who are not eligible for thrombolysis or thrombectomy.

In addition to efficacy, cost-effectiveness is also an important indicator for patients, physicians, and policy makers to determine whether the drug is widely used. To the best of our knowledge, no studies have explored the cost-effectiveness of tirofiban in patients with AIS. Therefore, the aim of this study is to evaluate the cost-effectiveness of tirofiban in AIS patients without large or medium-sized vessel occlusion from the perspective of healthcare systems in China and the US. The simulation spans a 20-year horizon.

## Materials and methods

This research adhered to the Consolidated Health Economic Evaluation Reporting Standards of 2022 (CHEERS 2022) protocol [18].

### Ethical approval from the committee

Ethical clearance from the institutional review board was not applicable as the study did not involve any human participants. The data analyzed in this research exclusively originated from publicly accessible resources and previously published papers.

### Participants

This research comprised a simulated cohort that adhered to the same inclusion criteria as observed in the RESCUE BT2 trial [17, 19]. In essence, the study encompassed ischemic stroke patients without large or medium -sized vascular occlusion, with approximately four distinct subgroups. The first group comprised patients who presented within 24 hours after the onset of stroke but were not eligible for intravenous or endovascular reperfusion therapy. The second group included patients who had not undergone reperfusion treatments due to patent proximal cerebral vessels, yet experienced progressive stroke symptoms between 24 to 96 hours after onset. The third and fourth groups encompassed patients who received IVT. The third group consisted of patients who encountered early neurological deterioration after treatment, while the fourth group consisted of patients who did not observe any neurological improvement post-treatment.

The baseline characteristics of the enrolled patients are available in S1 Table in S1 File.

### Intervention

**Fig 1:**
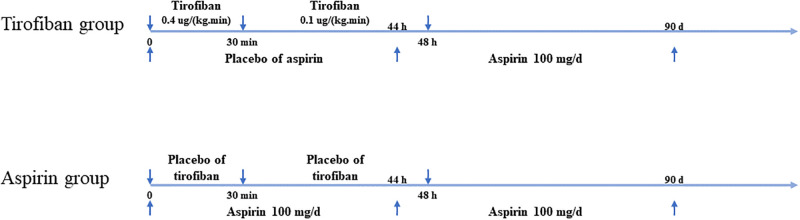
The switch of dosage and timing of tirofiban and aspirin throughout the trial. In the tirofiban group, patients received tirofiban at a dosage of 0.04 μg per min per kg after randomization, which was later reduced to 0.01 μg after 30 mins and continued for 48 hours. Concurrently, patients in this group were given a placebo of 100 mg aspirin per day. Conversely, in the aspirin group, the drug and placebo were switched. Aspirin was administered at a dosage of 100 mg at the 44th hour after randomization, lasting for 90 days in both groups.

The present study consisted of two distinct groups: the tirofiban group and the aspirin group. In the tirofiban group, intravenous tirofiban was introduced at a rate of 0.4 μg per kilogram of body weight every minute for a duration of 30 minutes. Following this initial phase, a constant infusion of 0.1 μg per kilogram per minute was maintained for a maximum of 48 hours. Patients who were part of the tirofiban group were concurrently administered a daily oral placebo for a span of 2 days. On the other hand, individuals in the aspirin group were designated to receive intravenous placebo alongside oral aspirin (100 mg per day) over the same 2-day period. From approximately the 44th hour after the intravenous tirofiban or placebo administration, all patients were prescribed oral aspirin at a daily dose of 100 mg, which continued until day 90. Fig 1 presents the dosage and timing of drugs administered throughout the trial.

### Model overview

We created a hybrid model using TreeAge Pro software 2020 (Williamstown, Massachusetts, USA), integrating a short-term decision tree and a long-term Markov model. This model enabled a comparative analysis of cost and effectiveness between tirofiban and aspirin in stroke patients without large or medium-sized vessel occlusion. During the initial 3 months, the cost and effectiveness in both groups were computed using the decision tree. In the subsequent cycles, the cost and effectiveness for both groups were evaluated using the Markov model. To determine the total cost per patient, we added up the costs incurred during the first 3 months and the subsequent cycles. The analysis was conducted over a time horizon of 20 years, which surpasses the life expectancy in both China and the US.

**Fig 2:**
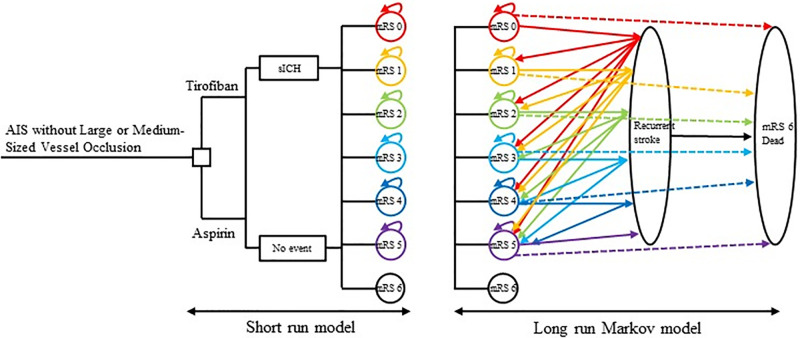
Schematic of the short and long run model. Patients in any mRS state could experience a recurrent stroke or non-stroke-related death. Those who experienced a recurrent stroke could die from it and would not be able to return to a lower mRS state. AIS: acute ischemic stroke. sICH: symptomatic intracranial hemorrhage. mRS: modified Rankin scale.

Prior to commencement, the patients were assigned randomly into two groups: the tirofiban group, receiving tirofiban along with a placebo, and the aspirin group, receiving aspirin alongside a placebo. At month 3, patients in each group exhibited different distributions on the mRS, and these proportions were directly derived from the RESCUE BT2 trial. This trial was a prospective, multi-center double-blind study that included 1158 Chinese patients. Following the initial 3-month period within the decision tree, patients progressed to the Markov model, with a cycle length of 3 months, starting with the mRS distribution obtained from the decision tree. The Markov model encompassed 7 health states representing distinct levels of disability: "mRS 0," "mRS 1," "mRS 2," "mRS 3," "mRS 4," "mRS 5," and "mRS 6 (Dead)." Importantly, it was assumed that patients experiencing a recurrent stroke during a Markov cycle could not transition to a lower disability level in the subsequent cycle, based on recommendations from neurologists. Additionally, it was essential to note that regardless of their mRS classification, all patients faced the possibility of mortality due to either stroke-related or non-stroke causes. For a visual representation of the potential transitions between Markov states, please refer to Fig 2.

As the most significant and serious adverse event (SAE), the incidence of symptomatic intracranial hemorrhage (sICH) in the tirofiban group was marginally higher than that observed in the aspirin group (Table 1). Although this information is not depicted in Fig 2, it was duly considered in our Markov model.

**Table 1:** Key input parameters of the Markov model in the study.

Parameters	Value	Distribution	Range/Parameters	Source
mRS distribution at month 3 in tirofiban				
mRS 0	0.106	Dirichlet	0–1	Reference [17]
mRS 1	0.185			
mRS 2	0.329			
mRS 3	0.219			
mRS 4	0.106			
mRS 5	0.017			
mRS 6	0.038			
mRS distribution at month 3 in aspirin				
mRS 0	0.083	Dirichlet	0–1	Reference [17]
mRS 1	0.139			
mRS 2	0.342			
mRS 3	0.291			
mRS 4	0.102			
mRS 5	0.016			
mRS 6	0.027			
Probability of sICH in tirofiban	0.01	β, SD 0.003	0–0.01	Reference [17]
Probability of sICH in aspirin	0	/		
Transition probability of recurrent stroke in China	0.017	β, SD 0.001	0.015–0.020	Reference [20, 21]
Transition probability of recurrent stroke in the US	0.013	β, SD 0.001	0.010–0.015	Reference [22, 23]
Death after recurrent stroke in China	0.21	β, SD 0.011	0.189–0.232	Reference [24, 25]
Death after recurrent stroke in the US	0.19	β, SD 0.051	0.1–0.3	Reference [24]
Death hazard ratios in China/US				
mRS 0	1	Lognormal, SD 0.050	1–1.2	Reference [22, 24]
mRS 1	1	Lognormal, SD 0.050	1–1.2	
mRS 2	1.11	Lognormal, SD 0.103	1–1.3	
mRS 3	1.27	Lognormal, SD 0.125	1.02–1.52	
mRS 4	1.71	Lognormal, SD 0.170	1.37–2.05	
mRS 5	2.37	Lognormal, SD 0.235	1.9–2.84	
Utilities in China				
mRS 0	0.95	β, SD 0.005	0.94–0.96	Reference [26]
mRS 1	0.89	β, SD 0.023	0.87–0.96	
mRS 2	0.67	β, SD 0.074	0.54–0.83	
mRS 3	0.44	β, SD 0.079	0.29–0.60	
mRS 4	0.16	β, SD 0.036	0.09–0.23	
mRS 5	0.1	β, SD 0.054	0–0.21	
mRS 6	0	/		
Stroke recurrence	0.42	β, SD 0.153	0.11–0.71	Reference [26]
Disutility of ICH	0.38	β, SD 0.041	0.30–0.46	Reference [24]
Utilities in the US				
mRS 0	0.85	β, SD 0.051	0.8–1	Reference [22, 27]
mRS 1	0.8	β, SD 0.038	0.8–0.95	
mRS 2	0.7	β, SD 0.055	0.68–0.9	
mRS 3	0.51	β, SD 0.050	0.45–0.65	
mRS 4	0.3	β, SD 0.075	0.1–0.4	
mRS 5	0.15	β, SD 0.080	0–0.32	
mRS 6	0	/		
Stroke recurrence	0.31	β, SD 0.033	0.24–0.37	
Disutility of sICH	0.38	β, SD 0.040	0.3–0.46	Reference [24]
Discount rate in China	0.05	/	0–0.08	Reference [28]
Discount rate in the US	0.03	/	0–0.08	Reference [22, 24]
Cost of tirofiban in China (CNY per mg)	37.2	γ, SD 3.72	36.24–48.84	Local data
Patient weight in China (kg)	75	β, SD 12.5	50–120	
IV infusion within 1h in China (CNY)	15.6	γ, SD 1.56	5–30	
IV infusion, additional 1h in China (CNY)	1	γ, SD 0.1	0.5–2	
Cost of tirofiban in the US (USD per mg)	19.29	γ, SD 1.93	9.64–38.57	Local data
Patient weight in the US (kg)	90	β, SD 15	60–150	
IV infusion within 1h in the US (USD)	142.55	γ, SD 14.3	100–200	
IV infusion, additional 1h in the US (USD)	30.68	γ, SD 3.07	20–40	
Other cost in China (CNY)				
Acute stroke (mRS 0–1)	12,336	γ, SD 2102	7126–15,533	Reference [24]
Acute stroke (mRS 2–5)	16,311	γ, SD 3106	8964–21,389	
Acute stroke (death)	13,979	γ, SD 2977	6568–18,476	
sICH	2979	γ, SD 1393	647–6217	
Annual posthospitalization (mRS 0–1)	8771	γ, SD 2140	2626–11,188	
Annual posthospitalization (mRS 2–5)	13,345	γ, SD 3356	3356–16,783	
Recurrent stroke	18,180	γ, SD 2272	13,635–22,726	
Other cost in the US (USD)				
Acute stroke (mRS 0–2)	15,561	γ, SD 1556	15,375–15,748	Reference [22]
Acute stroke (mRS 3–5)	19,345	γ, SD 1935	19,108–19,581	
Acute stroke (death)	25,425	γ, SD 2543	24,469–26,382	
sICH	3678	γ, SD 368	2942–4414	
Quarterly posthospitalization (mRS 0)	3069	γ, SD 307	2455–3682	
Quarterly posthospitalization (mRS 1)	2966	γ, SD 297	2528–3791	
Quarterly posthospitalization (mRS 2)	3655	γ, SD 366	2925–4386	
Quarterly posthospitalization (mRS 3)	6277	γ, SD 628	5022–7532	
Quarterly posthospitalization (mRS 4)	12,705	γ, SD 1271	10,163–15,246	
Quarterly posthospitalization (mRS 5)	18,678	γ, SD 1868	14,942–22,413	
Recurrent stroke	22,274	γ, SD 2227	21,590–23,034	
Background mortality in China				
68–69 years old	0.01266	/		Reference [29]
70–74	0.02159			
75–79	0.03731			
80–84	0.06340			
85–89	0.15120			
Background mortality in the US	S1 File			

mRS, modified Rankin Scale; sICH, symptomatic intracranial hemorrhage; IV, intravenous; CNY. Chinese Yuan;

### Transition probabilities

The distributions of mRS at month 3 were acquired directly from the RESCUE BT2 trial and are presented in Table 1.

In the Markov model, it was assumed that the prognosis of patients who survived beyond 3 months solely depended on the level of disability, namely the mRS classification, not depended on whether tirofiban or aspirin was given when enrolled in the study at the beginning. The incidence rate of recurrent stroke was obtained from the domestic data in China and US, and the mortality rate post recurrent stroke was a bit higher in China than in the US, which was 0.21 and 0.19, [22, 30–32] respectively. For non-stroke caused death, considering that disabling patients were prone to death than the general population with same age, a hazard ratio was employed to adjust the non-stroke caused mortality rate. The background mortality was accessed from the healthcare yearbook launched by the government. In addition, patients who survived after experiencing a recurrent stroke were assumed to be evenly distributed among health states with comparable or higher levels of disability.

The annual incidence rate (R) was transformed into a 3-month incidence rate (r) using the formula "r = -ln (1—R)/4," and subsequently, the 3-month transition probability (p) was computed with the formula "p = 1—exp(-r)" [33, 34].

### Costs

The investigation was conducted from the perspective of the healthcare system, focusing exclusively on direct costs and excluding any indirect costs or direct non-medical costs. To accurately represent the costs in 2022, all costs not priced in that year were adjusted using the Healthcare Consumer Price Index. Additionally, the study also accounted for future costs by applying a discount rate of 0.05 in China, within an interval ranging from 0 to 0.08, and a discount rate of 0.03 in the US.

The cost of tirofiban in China varies among manufacturers. For our study, we used the cost from the RESCUE BT2 trial, which was 37.2 CNY per mg. In contrast, the cost of tirofiban in the US was $19.29 per mg. Considering the notable difference in the average weight of Chinese patients compared to US patients, we assumed an average weight of 75 kg for Chinese patients and 90 kg for US patients. Weight ranges were set at 50–120 kg for Chinese patients and 60–150 kg for US patients. Furthermore, there are significant variations in the costs of intravenous infusions between China and the US, and we have included these costs in our analysis.

Stroke-related costs encompassed three main aspects, namely the cost of the acute phase, which involved hospitalization expenses, the annual cost of post-hospitalization care, and the cost associated with recurrent stroke occurrences. The stroke-related costs vary with different mRS classifications.

The cost of the acute phase of stroke and the annual post-hospitalization care for stroke in China was derived from the China National Stroke Registry (CNSR) and inflated to reflect the costs in China in 2022. The cost of recurrent stroke in China was obtained from a published paper that reported the cost within their institution. Additionally, the cost of sICH in China was obtained from the database of Thrombolysis Implementation and Monitor of Acute Ischemic Stroke in China (TIMS-China). The stroke-related cost and the cost of sICH in the US were obtained from a study investigating the cost-effectiveness of mechanical thrombectomy for stroke in the US setting, and both were higher than their counterparts in China [22]. In the US, the cost of stroke treatment varied based on different mRS classifications, exhibiting higher costs with more severe mRS classifications. This trend held true for both the acute phase and post-hospitalization care [22].

### Utility

In the present study, the effectiveness was calculated by multiplying the number of life years with the utility of the corresponding mRS classifications, measured with quality-adjusted life year (QALY). Additionally, future effectiveness would be discounted using the same discount rate as applied to the costs.

Various utilities were employed for different mRS classifications, as higher mRS classifications indicated a higher level of disability. Additionally, different utilities were allocated to the same mRS classification for patients in China and the US, with each country adopting utilities relevant to their respective settings.

Disutilities were applied to the events of recurrent stroke and sICH, as these events result in a decreased quality of life.

### Outcomes

The study focused on several key outcomes: respective costs, effectiveness in both groups, and the incremental cost-effectiveness ratio (ICER) of tirofiban versus aspirin. Tirofiban’s cost-effectiveness was determined based on whether the obtained ICER fell within the willing-to-pay (WTP) threshold. In China, the WTP threshold was 85,698 CNY per QALY, while in the US, it was $100,000 per QALY. If the ICER was below the threshold, tirofiban was considered cost-effective; if it exceeded the threshold, tirofiban was deemed not cost-effective.

### Sensitivity analysis

To ensure the robustness of our findings, we conducted both one-way sensitivity analysis and probabilistic sensitivity analysis. In one-way sensitivity analysis, input parameters were varied within their 95% confidence interval or specified range, and the results were visually presented using a Tornado diagram, highlighting the impact of each parameter on the outcomes. For probabilistic sensitivity analysis (PSA), we performed 10,000 iterations of sampling to assess result uncertainty. All cost parameters followed a gamma distribution, while transition probabilities and utility parameters followed a beta distribution. For mRS classifications at month 3, it followed the Dirichlet distribution. A cost-effectiveness plane and an acceptability curve were used to illustrate the outcomes. Furthermore, to ensure the robustness of our findings across diverse populations, we conducted subgroup analyses across various age groups and genders, considering the specific background mortality rates within each population.

## Results

### Base case analysis

For AIS without large or medium-sized vascular occlusion in China, the total cost was 101,662 CNY when tirofiban was administered alongside standard treatment, while it would be 103,803 CNY if aspirin plus standard treatment was used. The corresponding effectiveness was 3.62 QALYs and 3.48 QALYs, respectively. The ICER was calculated to be -15,197 CNY per QALY (Table 2).

**Table 2:** The main results of costs and effectiveness in both China and the US.

	Total cost	Total eff*	Incr-cost	Incr eff*	ICER
**China (Cost united in CNY)**					
Aspirin	103,803	3.48	/	/	/
Tirofiban	101,662	3.62	-2141	0.14	-15,197
**US (Cost united in USD)**					
Aspirin	201,984	4.06	/	/	/
Tirofiban	197,055	4.15	-4929	0.08	-58,296

CNY. Chinese Yuan; eff, effectiveness; Incr, incremental; ICER, incremental cost-effectiveness ratio.

* The effectiveness was united in quality-adjusted life year.

In the US settings, tirofiban still exhibited a lower cost compared to aspirin ($197,055 versus $201,984) and demonstrated higher effectiveness (4.15 QALYs versus 4.06 QALYs), resulting in an ICER of -$58,296 per QALY (Table 2).

### One-way sensitivity analysis

**Fig 3:**
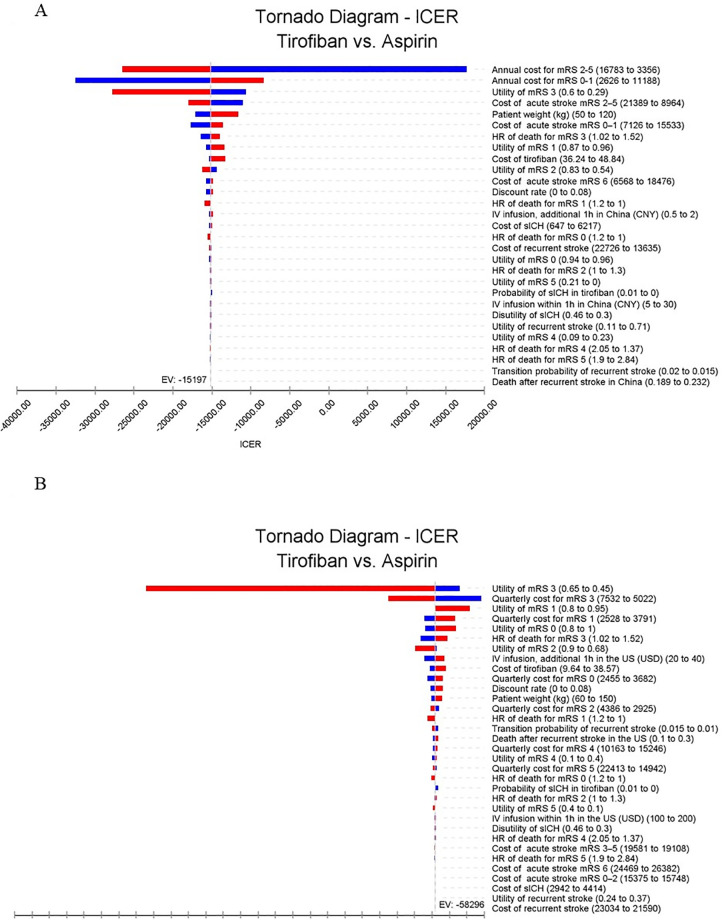
Tornado diagram depicting the impact of each parameter on the ICER. Red represents the upper range of the parameter, while blue corresponds to the lower range of the parameter. Fig 3A displays the Tornado diagram of ICER in the China setting, while Fig 3B illustrates the Tornado diagram of ICER in the US setting. ICER denotes incremental cost-effectiveness ratio. In both China and the US, the mRS utility and the annual cost of post-hospitalization care had the most significant impact on the ICER. ICER: incremental cost-effectiveness ratio. mRS: modified Rankin scale. HR: hazards ratio. IV: intravenous. sICH: symptomatic intracranial hemorrhage.

In the one-way sensitivity analysis, the annual cost of post-hospitalization stroke care had the most significant impact on the ICER in Chinese patients (Fig 3A). However, even with the highest post-hospitalization costs, the ICER remained below 85,698 CNY per QALY. On the other hand, in the US settings, the utility of mRS 3 had the most substantial influence on the ICER, yet it did not lead to an ICER greater than 0 (Fig 3B). It is important to highlight that in both Chinese and US settings, key parameters, such as the cost of tirofiban, acute stroke treatment, post-stroke care, and utilities for various mRS classifications, did not result in an ICER surpassing the WTP threshold.

### PSA

The PSA results were depicted using a cost-effectiveness plane and an acceptability curve for cost-effectiveness In the cost-effectiveness plane, it is evident that all the data points lie below the WTP threshold line, regardless of whether in Chinese or US settings (S1 Fig in S1 File). Notably, over 95% of these data points are situated in the fourth quadrant. Analyzing the cost-effectiveness acceptability curve (S2 Fig in S1 File), we observe that when the WTP threshold was set to 0, tirofiban exhibited an acceptability rate of over 95% for both Chinese and US stroke patients.

### Subgroup analysis

As evident in S3, S4 Tables in S1 File, subgroup analyses across diverse age groups and genders in both China and the US indicated that, irrespective of patient age or gender, tirofiban demonstrated increased effectiveness at a lower cost in AIS patients without large or medium-sized vessel occlusion.

## Discussion

Our study indicated that in both China and the US, the use of tirofiban was associated with lower overall costs and higher effectiveness than aspirin in AIS patients without large or medium-sized vessel occlusion, resulting in an ICER of -15,197 CNY and -$58,296 per QALY, respectively. The robustness of our conclusion that tirofiban is cost-effectiveness or even cost-saving is supported by sensitive analyses. When the input parameters varied in corresponding ranges, the ICERs were below the WTP threshold both in China and the US.

The main reason why tirofiban is cost-saving lie in that it improves the degree of disability (evaluated by the mRS score), so that the cost of nursing and hospitalization are reduced. Meanwhile, as the degree of disability improved, the life quality of patients is increased and eventually the QALY is higher. Although a slightly higher death rate was observed in tirofiban group than aspirin group (3.8% vs. 2.6%) in RESCUE BT2 trial, [17] the benefits from significant improvement of disability offsets the adverse effect caused by death. Our one-way sensitive analyses confirmed this, the ICERs were observed to be most sensitive to annual cost for disabling stroke (mRS 2 to 5) in China and to utility of mRS 3 in the US, respectively, which illustrated that the improvement of mRS leads to the advantage of tirofiban in both cost and efficacy. In addition, PSA showed tirofiban had an over 95% probability of being cost-effective in China and the US after 10 000 iterations. Therefore, tirofiban is a dominant strategy compared with aspirin for patients with AIS without large or medium-sized vessel occlusion.

We evaluated the cost-effectiveness of tirofiban in patients with AIS in China and the US mainly because of the huge burden of stroke in those two countries. There were 3.94 million new stroke cases, 28.76 million prevalent cases and 2.19 million deaths due to stroke in China according to the data from GBD 2019 [4]. Additionally, stroke is the third leading cause of death and the highest cause of DALYs in China. While in the US, about 795 000 individuals in the US experience a new or recurrent stroke, of which 87% (690 000) are ischemic and 185 000 are recurrent [35]. The deaths due to stroke accounted for 5.2% of all deaths in the US in 2017, making it the most burdensome neurological disorder [36]. Despite great efforts in stroke treatment in recent years, the age-standardized mortality rates decreased by 39.8% in China from 1990 to 2019 and by 64.7% in women and 65.5% in men in the US from 1975 to 2019, respectively, the burden of stroke in China and the US is still severe [36, 37]. Therefore, new therapies are warranted to improve the outcomes of stroke patients and expand their life and QALY.

Reperfusion therapies, including IVT or EVT, are the main ways to promote cerebral artery blood flow recovery [7]. However, only a small proportion of patients are eligible for the indication, and the remaining patients can only be treated with aspirin or other drugs for antiplatelet therapy in the acute phase, which is far less beneficial than reperfusion therapy [19, 38]. Tirofiban, which could reversibly inhibit fibrinogen dependent platelet aggregation and subsequent thrombosis, has been shown to have favorable efficacy in improving vascular recanalization and long-term functional outcome for stroke in observational studies, but it has not shown significant benefits in stroke patients in previous clinical trials [14–16, 39–41]. The Study of Efficacy of Tirofiban in Acute Ischemic Stroke (SETIS) trial, which enrolled 150 AIS patients (within 6-hour window) who were not eligible for thrombolysis, showed that tirofiban was safe but was stopped early at the interim analysis due to lack of efficacy and low recruitment rate [40]. The Safety of Tirofiban in acute Ischemic Stroke (SaTIS) trial, which included 260 patients with AIS (within 48-hour window) and used placebo as a control, showed similar results to the SETIS trial [41]. The lack of a benefit of tirofiban in these two studies may be due to the small sample size and the use of efficacy outcomes as secondary outcomes in the SaTIS study. Later, the Efficacy and Safety of Tirofiban in Clinical Patients with acute Ischemic Stroke (ESCAPIST) trial recruited 380 patients with mild-to-moderate stroke (within 12-hour window) and found that patients in tirofiban group were more likely to have better functional outcomes than patients in aspirin group [16]. More recently, RESUE BT2, enrolled a broader population of patients with stroke of recent onset or progression of stroke symptoms and nonoccluded large and medium-sized cerebral vessels, demonstrated that using tirofiban can improve the mRS score among them [17]. However, the large-scale use of tirofiban in clinical practice depends not only on the efficacy but also on the cost-effectiveness. As the RESUE BT2 trial was the largest trial (included 1117 patients) to demonstrate the efficacy of tirofiban in AIS patients in the current and included a broader stroke population, we evaluated the cost-effectiveness of tirofiban based on the results of that trial. Our study further demonstrate tirofiban is cost-saving and can be a dominant strategy in these patients. Nonetheless, as this study was based on the results of only one large clinical trial, the cost-effectiveness analysis of tirofiban in AIS patients should be reevaluated when more future clinical trials prove the efficacy of tirofiban in AIS.

Our study has some limitations. First, our study was based on the efficacy findings of RESCUE BT2 trial which conducted in China, it is not clear whether tirofiban has the same effect in other populations. Additionally, the enrolled patients in the trial were highly selective and the improvement in mRS of tirofiban may varied in real-word population, therefore, generalizing our findings to other situations should be done with caution. Second, the input parameters we used were from previously published literatures that may be inaccurate and heterogeneous, thus causing bias to our results. Third, we assumed future changes in health status result from stroke and all cause of deaths in Markov model, but the transitions of health status as a result from other causes were not considered. Forth, in our Markov model, we assumed that patients with recurrent strokes would transition to the same or higher mRS classifications, and those without recurrent strokes would maintain the same classification in the subsequent cycle. Contrary to our assumptions, evidence suggests that all these patients are likely to transition to lower mRS classifications. Numerous studies highlight the potential for disability improvements or mRS classification changes, even among patients with recurrent strokes or up to one year after the initial stroke onset [42, 43]. This suggests that our model might, to some extent, lead to an overestimation of the cost of care. Although there are some limitations among the study, as the findings were robust in varied sensitive analyses, the overall of our results were unlikely be affected. Finally, our study, conducted from a healthcare system perspective, focused solely on direct medical costs. We did not incorporate indirect costs or direct non-medical costs in our analysis. A comprehensive societal perspective study, encompassing all potential costs, may provide a more accurate assessment of tirofiban’s cost-effectiveness.

## Conclusion

Treatment with tirofiban in AIS patients without large or medium-sized vessel occlusion is cost-saving in China and the US. Further studies are needed to demonstrate its cost-effectiveness in other populations and countries.

## Supporting information

S1 File (DOC)

S1 Checklist (DOCX)
