# Supplementary material for: Cost-effectiveness of tirofiban for acute ischemic stroke without large or medium-sized vessel occlusion: A Markov modelling analysis from the Chinese and United States perspectives
Source: PLoS One. 2024 Feb 16;19(2):e0297939. doi: 10.1371/journal.pone.0297939 (PMC10871502; doi:10.1371/journal.pone.0297939)
Supplement: S1 File — (DOC) [file pone.0297939.s001.doc]

**S1 Table: Baseline Characteristics of Enrolled Patients in the RESCUE BT2 Study**

| **Variables** | **Value** |
| --- | --- |
| Total patients-n. | 1177 |
| Median age-yr | 68 |
| Male sex-% | 63.9 |
| Clinical history-% |  |
| Cerebral infarction | 15.2 |
| History of antiplatelet use | 20.5 |
| History of anticoagulation | 0.1 |
| Median NIHSS score (IQR) | 9.0 (7.0–10.0) |
| Median ASPECTS (IQR) | 9.0 (9.0–10.0) |
| Median systolic blood pressure at hospital arrival-mm Hg | 155 |
| Median glucose level at hospital arrival | 6.5 |
| Presentation type-% |  |
| Ineligible for reperfusion treatment and within 24 hr after stroke onset | 55.2 |
| Ineligible for reperfusion treatment and progression 24–96 hr after stroke onset | 32.2 |
| IVT followed by early neurologic deterioration | 7.6 |
| IVT followed by no neurologic improvement | 4.9 |
| Localization of presenting deficit-% |  |
| Anterior circulation | 80.3 |
| Posterior circulation | 15.8 |
| Anterior circulation plus posterior circulation | 1.0 |
| Unknown | 2.9 |
| Presumed mechanism of ischemic cerebral event-% |  |
| Artery-to-artery embolism | 9.0 |
| Hypoperfusion or impaired emboli clearance beyond a stenosis | 4.7 |
| Penetrating artery disease | 72.6 |
| In situ thrombo-occlusion distal to a stenosed artery | 1.4 |
| Mixture of the above | 7.6 |
| Unknown | 3.8 |
| Median time from stroke onset or progression of stroke symptoms to randomization (IQR)-hr | 11.0 (7.2–16.8) |
| Median time from stroke onset or progression of stroke symptoms to  initial treatment (IQR)-hr | 11.4 (7.5-17.1) |

Abbreviations: IQR, interquartile range; IVT, intravenous thrombolysis.

**S2 Table: Background mortality rate in the US**

| **Age** | **Background mortality rate** | **Source** |
| --- | --- | --- |
| 68 | 0.018272 | https://www.cdc.gov/nchs/products/life_tables.htm |
| 69 | 0.019676 |
| 70 | 0.021199 |
| 71 | 0.022881 |
| 72 | 0.024832 |
| 73 | 0.026725 |
| 74 | 0.030032 |
| 75 | 0.032663 |
| 76 | 0.036297 |
| 77 | 0.039811 |
| 78 | 0.044410 |
| 79 | 0.048780 |
| 80 | 0.053900 |
| 81 | 0.059479 |
| 82 | 0.065797 |
| 83 | 0.073678 |
| 84 | 0.082232 |
| 85 | 0.091981 |
| 86 | 0.100813 |
| 87 | 0.113298 |
| 88 | 0.126982 |
| 89 | 0.141894 |
| 90 | 0.158045 |


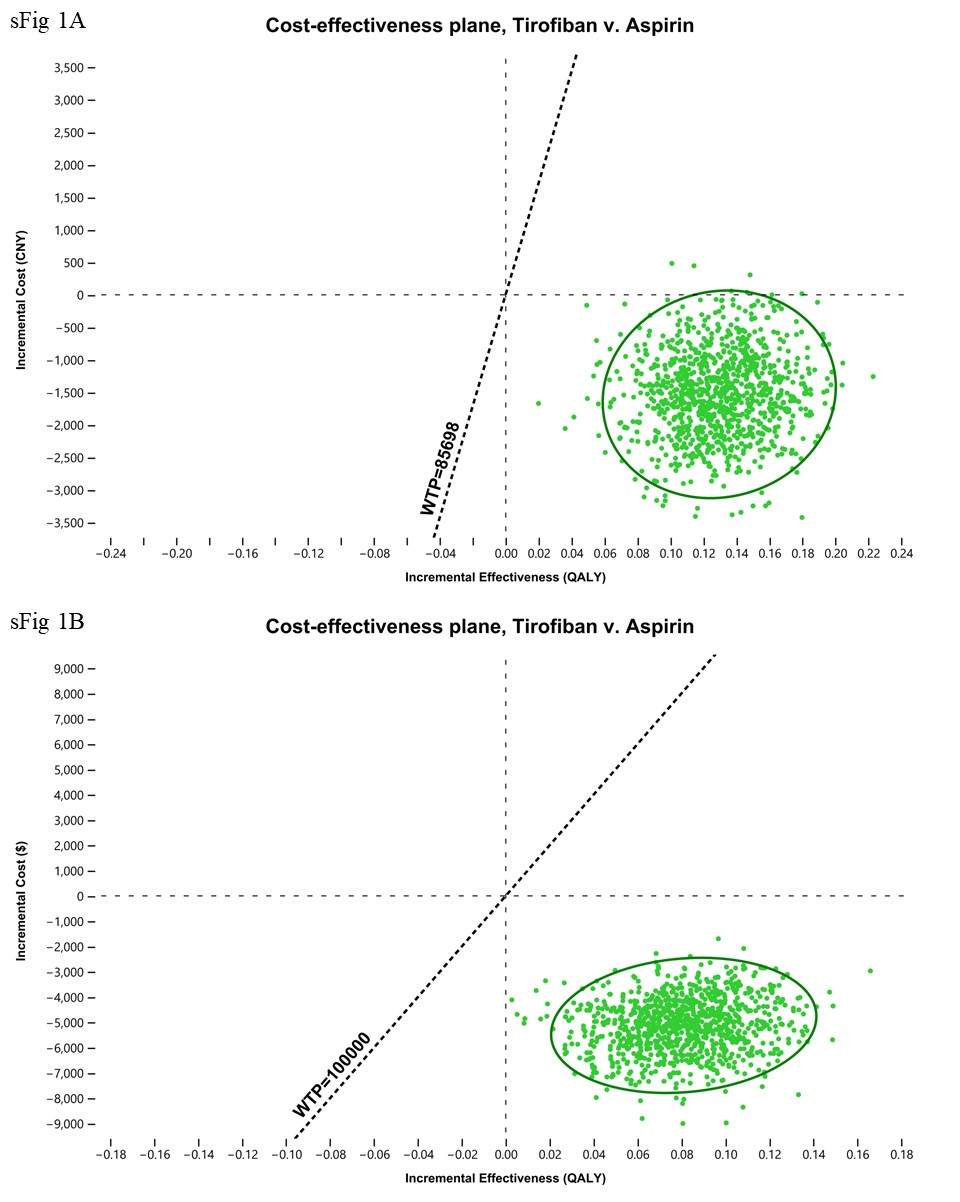


**S1 Fig.** **Cost effectiveness plane of tirofiban versus aspirin.** Figures 1A and 1B show the cost-effectiveness planes of tirofiban versus aspirin in China and the US, respectively. In both settings, nearly all data points fall below the willingness-to-pay threshold line.


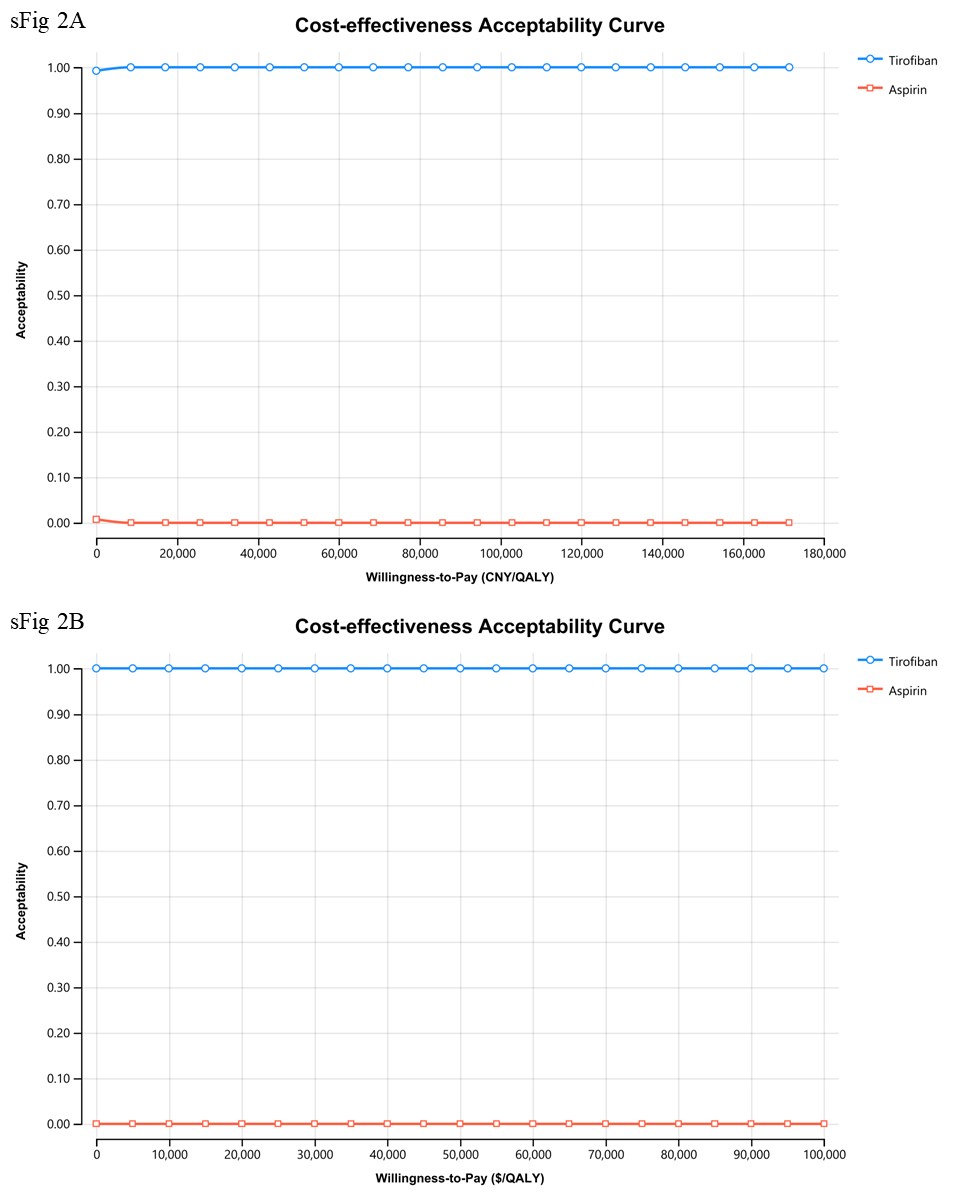


**S2 Fig. Cost effectiveness acceptability curve of tirofiban and aspirin.** Figures 1A and 1B depict the acceptability of tirofiban and aspirin in China and the US, respectively. It is evident that the acceptability of tirofiban was consistently higher than that of aspirin across different willingness-to-pay thresholds and in both China and the US.

**S3 Table: Subgroup Analysis Across Different Ages and Genders in China**

|  | **Total cost** | **Total eff*** | **Incr-cost** | **Incr eff*** | **ICER** |
| --- | --- | --- | --- | --- | --- |
| **A** **60-year-old Chinese male (Cost presented in CNY)** | | | | | |
| Aspirin | 107,461 | 3.59 | / | / | / |
| Tirofiban | 105,183 | 3.74 | -2277 | 0.14 | -15,849 |
| **A 60-year-old Chinese female (Cost presented in CNY)** | | | | | |
| Aspirin | 110,906 | 3.71 |  |  |  |
| Tirofiban | 108,498 | 3.85 | -2409 | 0.15 | -16,469 |
| **A 70-year-old Chinese male (Cost presented in CNY)** | | | | | |
| Aspirin | 98,071 | 3.28 |  |  |  |
| Tirofiban | 96,146 | 3.42 | -1925 | 0.14 | -14141 |
| **A 70-year-old Chinese female (Cost presented in CNY)** | | | | | |
| Aspirin | 104,232 | 3.49 |  |  |  |
| Tirofiban | 102,075 | 3.63 | -2157 | 0.14 | -15271 |

**S4 Table: Subgroup Analysis Across Different Ages and Genders in the US**

|  | **Total cost** | **Total eff*** | **Incr-cost** | **Incr eff*** | **ICER** |
| --- | --- | --- | --- | --- | --- |
| **A 60-year-old US male (Cost presented in USD)** | | | | | |
| Aspirin | 210,145 | 4.19 | / | / | / |
| Tirofiban | 204,906 | 4.28 | -5239 | 0.09 | -61,457 |
| **A 60-year-old US female (Cost presented in USD)** | | | | | |
| Aspirin | 219,179 | 4.43 |  |  |  |
| Tirofiban | 213,596 | 4.34 | -5583 | 0.09 | -64,989 |
| **A 70-year-old US male (Cost presented in USD)** | | | | | |
| Aspirin | 190,129 | 3.86 |  |  |  |
| Tirofiban | 185,655 | 3.95 | -4474 | 0.08 | 53,710 |
| **A 70-year-old US female (Cost presented in USD)** | | | | | |
| Aspirin | 202,947 | 4.08 |  |  |  |
| Tirofiban | 197,981 | 4.16 | -4966 | 0.08 | -58680 |
